# Pathobiomes Differ between Two Diseases Affecting Reef Building Coralline Algae

**DOI:** 10.3389/fmicb.2017.01686

**Published:** 2017-09-01

**Authors:** Anne-Leila Meistertzheim, Maggy M. Nugues, Gaëlle Quéré, Pierre E. Galand

**Affiliations:** ^1^CNRS, Laboratoire d’Ecogéochimie des Environnements Benthiques (LECOB), Observatoire Océanologique, Sorbonne Universités, UPMC Univ Paris 06 Banyuls-sur-Mer, France; ^2^EPHE-UPVD-CNRS, USR 3278 CRIOBE, Université de Perpignan, PSL Research University Perpignan, France; ^3^Laboratoire d’Excellence “CORAIL” Moorea, French Polynesia

**Keywords:** disease, bacterial communities, crustose coralline algae, bacterial pathogen, causative agent, coral reefs

## Abstract

Crustose coralline algae (CCA) are major benthic calcifiers that play crucial roles in coral reef ecosystems. Two diseases affecting CCA have recently been investigated: coralline white band syndrome (CWBS) and coralline white patch disease (CWPD). These diseases can trigger major losses in CCA cover on tropical coral reefs, but their causative agents remain unknown. Here, we provide data from the first investigation of the bacterial communities associated with healthy and diseased CCA tissues. We show that *Neogoniolithon mamillare* diseased tissues had distinct microbial communities compared to healthy tissues and demonstrate that CWBS and CWPD were associated with different pathobiomes, indicating that they had different disease causations. CWBS tissues were composed of opportunistic bacteria, and the origin of the disease was undetermined. In contrast, a vibrio related to *Vibrio tubiashii* characterized the CWPD pathobiome, suggesting that it could be a putative disease agent and supporting the case of a temperature dependent disease associated with global warming.

## Introduction

In recent decades, there has been an increasing number of reports of diseases in marine organisms, such as corals, mollusks, turtles, and mammals (Harvell et al., 1999, 2004). Infectious diseases have been acknowledged as a major factor of change in marine ecosystems. They can lead to a loss of keystone species and alter critical ecosystem processes (Harvell et al., 2002). However, their causal agents often remain unknown, thus slowing the development of management and remediation tools (Harvell et al., 2004). Together with scleractinian corals, crustose coralline algae (CCA) are major framework builders and carbonate producers on tropical reefs (Rasser and Riegl, 2002). Two disease symptoms have been recently investigated: the coralline white band syndrome (CWBS) and the coralline white patch disease (CWPD) (Quéré et al., 2015b). White band lesions are defined by bands that appear centrally or peripherally and advance on healthy tissue, whereas the patch disease shows distinct white patches expanding on healthy crusts (Quéré et al., 2015a). Both diseases result in tissue loss, which often leads to the death of the affected patch, with subsequent colonization by endophytic algae (Quéré et al., 2015b). Although these syndromes have been well-described in field surveys, standard histopathological techniques have failed to identify the potential infectious agents (Quéré et al., 2015a). Further molecular characterization of the microbial communities is needed to complete the diagnostic picture.

The microbes that are associated with macro-organisms play an important role in the host’s health and metabolism (Rosenberg and Zilber-Rosenberg, 2016). Microbial communities associated with CCA remain poorly understood (Webster et al., 2011; Sneed et al., 2015; Hester et al., 2016), but there are indications that they can induce settlement and metamorphosis of coral larvae (Negri et al., 2001). CCA bacterial communities can be altered by environmental changes such as temperature (Webster et al., 2011), pH (Webster et al., 2013), or disease (Kimes et al., 2010), but the identification of the causative pathogens associated with epizootics in CCA is still unknown. Recently, the development of “omic” studies has shown the limits of the Koch and Hill’s postulate “one microbe – one disease” and brought forward the pathobiome concept (Vayssier-Taussat et al., 2014), which has been proposed for the study of coral diseases (Sweet and Bulling, 2017). The pathobiome is defined as the pathogenic agent integrated within its biotic environment (Vayssier-Taussat et al., 2014). Pathogens interact with other microbes, and these complex interactions influence the outcome of the disease.

Here, we investigated both healthy and diseased CCA tissues to test the hypothesis that tissue lesions indicative of CWBS and CWPD are characterized by distinct pathobiomes.

## Materials and Methods

### Field Collection

**FIGURE 1:**
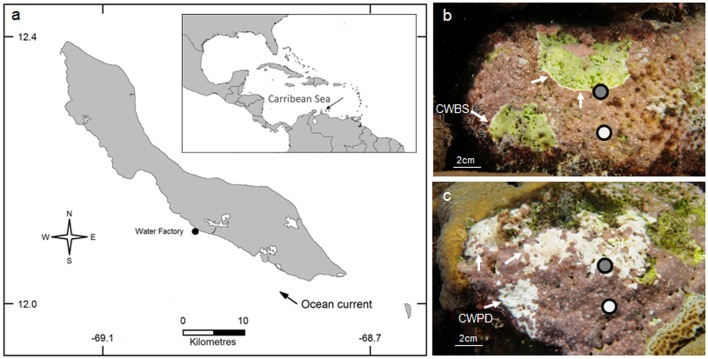
Geographical location of the Water Factory sampling site in Curaçao in 2014. The dark gray areas indicate urbanized area **(a)**. *Neogoniolithon mamilla*re affected by coralline white band syndrome (CWBS) **(b)** and coralline white patch disease (CWPD) **(c)**. Healthy and diseased tissues sampled for each pathology are indicated in white and gray shading respectively. Arrows point to the white bands in CWBS and the white patches in CWPD.

The common CCA species *Neogoniolithon mamillare* was sampled on 25 October and 15 November 2014 at the diving site Water Factory on the leeward coast of Curaçao, Southern Caribbean (12°N, 69°W) (**Figure 1a**). Fragments (ca. 2–5 cm^2^) of the CCA species were collected using hammer and chisel on the reef terrace at 5–10 m depth. Two different symptoms were targeted: CWBS and the CWPD (**Figures 1b,c**). Samples were gathered from five healthy-looking individuals, five CWBS and five CWBD individuals. Fragments of diseased individuals included both healthy-looking and diseased tissues. Healthy and diseased individuals were placed in individual collection bags to avoid contamination and transported in the dark to the laboratory.

### DNA Extraction and Sequencing

Back in the laboratory, the CCA fragments were dissected with sterilized instruments by scraping the surface of the samples. One sample was chipped from each of the five healthy-looking individuals (controls), whereas a sample of healthy-looking tissue and a sample of diseased-looking tissue was chipped from each of the 10 diseased individuals, making a total of 25 samples. Samples of diseased-looking tissue included the boundary between healthy and diseased tissues (**Figures 1b,c**). Each sample was fixed separately in ethanol 96% v/v. Lysis of CCA was realized by mechanic action on a FastPrep Instrument with a Y Matrix (MP Biomedical, Santa Ana, CA, United States), after removing the ethanol and washing with DNase free water, followed by incubation with proteinase K at 57°C during 1 h. DNA was extracted using Maxwell^®^ Blood DNA Purification Kit LEV (Promega, Madison, WI, United States) on Maxwell 16 MDx Instrument (Promega) following the manufacturer instructions. DNA concentrations were measured by spectrophotometry (Nanodrop ND-1000, Thermo Fisher Scientific, Inc., Waltham, MA, United States), and the quality was assessed by electrophoresis migration on a 1% agarose gel. DNA was diluted to 15 ng μL^-1^ and 30 ng were used for PCR amplification. The V1–V3 region of the bacterial 16S rRNA genes were amplified using bacteria specific primers 27F (AGRGTTTGATCMTGGCTCAG) and 519R (GTNTTACNGCGGCKGCTG) with the barcode on the forward primer. All samples were then pooled together in equal proportions based on their DNA concentrations. Pooled samples were purified using calibrated Agencourt^®^ AMPure^®^ XP (Beckman Coulter, Brea, CA, United States). Then the pooled and purified PCR product was used to prepare a DNA library by following Illumina TruSeq DNA library preparation protocol. All samples were sequenced on the same Miseq Illumina sequencer run (Illumina, San Diego, CA, United States) using Miseq reagent kit V3 (Illumina) producing 2 × 300-bp long reads in a commercial laboratory (MR DNA, Lubbock, TX, United States). All sequences were deposited in GenBank under SRA accession SRP113196.

### Sequence Analyses

R1 and R2 reads were joined with PANDAseq (Masella et al., 2012). The read quality filtering and length trimming, data set partitioning based on barcodes, de-replication, clustering at 97% sequence identity and taxonomic classification were performed with PyroTagger (Kunin and Hugenholtz, 2010). The quality of the sequences was controlled by removing all the reads that had a mismatch with the 16S rRNA primers, contained ambiguous nucleotides (N) or were <300 bp long beyond the forward primer. A stringent quality trimming criteria was applied to remove reads that had ≥10% of bases with Phred values <27. This procedure is recommended to ensure that when clustering at 97%, the influence of erroneous reads is minimized (Kunin et al., 2010). The sequences were then de-replicated and clustered at a 97% threshold using UCLUST (Edgar, 2010). The sequences from each operational taxonomic unit (OTU) were classified by comparing them with those from the SILVA 119 (Quast et al., 2013). The taxonomic affiliations of the OTUs of interest were further verified against sequences from the NCBI databases using BLAST with default parameters. Sequences annotated as belonging to algal chloroplasts were removed. Putatively chimeric sequences were identified as sequences having a best Blast alignment <90% of the trimmed read length to the reference sequence in the database, ≥90% sequence identity to the best Blast match and OTU size ≤2. One sample from healthy patch disease tissues was dominated by a chimeric sequence and was thus removed from the dataset.

### Data Analysis

A total of 6,083,434 bacterial 16S rRNA genes sequences were retained after removing poor quality reads and putative algal chloroplastic hits. A total of 10,818 OTUs were identified at a 97% sequence similarity cutoff. All samples were randomly re-sampled to match the size of the sample containing the fewest sequences (*n* = 11,695). The Shannon diversity index (H′) was calculated at the OTU level on the re-sampled dataset with the PAST software (Hammer et al., 2001). Differences in OTU abundance between two groups of samples were tested using the White’s non-parametric *t*-test using the STAMP v2.1.3 software (Parks et al., 2014). OTUs were defined as pathology-specific when their representative sequence abundance was significantly higher in the diseased tissues compared to control tissue.

A multidimensional scaling ordination (MDS) based on Bray–Curtis similarity was conducted to visualize similarities in community composition between samples. The MDS was computed with the R package phyloseq (McMurdie and Holmes, 2013). An analysis of similarity (one-way ANOSIM) was conducted to assess the significance of the MDS grouping using PAST v3.10 software. A *t*-test was used to assess the differences in community diversities were significant using Statistica v10 software after testing for normality and equal variance.

To test if *N. mamillare* had a species specific bacterial community, OTU sequence representatives were blasted against 454 sequences previously found in four different species of CCA sampled in Belize: *Titanoderma prototypum*, *Porolithon pachydermum*, *Paragoniolithon solubile*, and *Hydrolithon boergesenii* (SRA database number SRP056487) (Sneed et al., 2015).

## Results and Discussion

**FIGURE 2:**
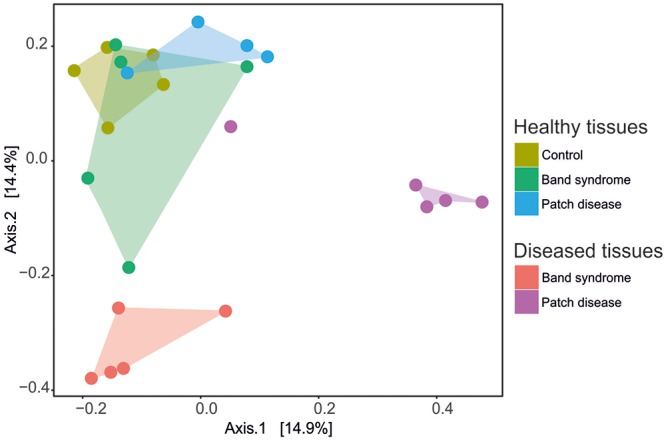
Multi-dimensional scaling plot (MDS) based on the Bray–Curtis similarity index showing the similarity between bacterial community compositions for healthy and diseased tissues in CCA affected by CWBS and CWPD.

**FIGURE 3:**
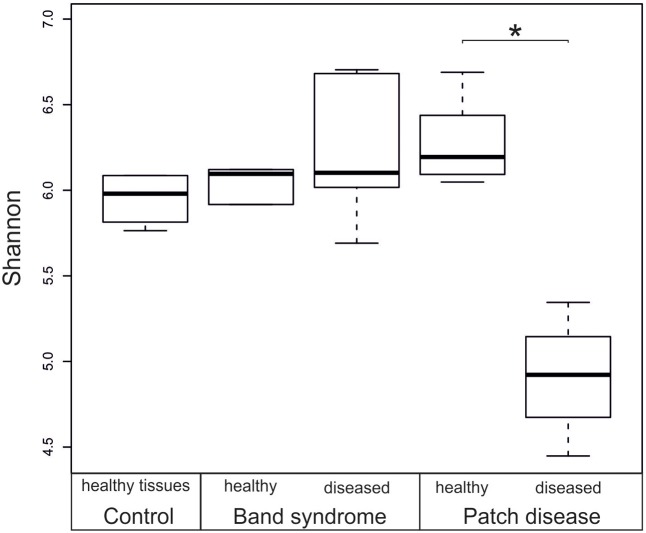
Box plot showing the diversity (Shannon index) based on 16S rRNA sequences from healthy and diseased *N. mamillare* tissues. The mean value and the standard deviation are represented by a line and a box respectively, and minimum and maximum values are represented by whiskers. ^∗^*p* < 0.05.

A comparison of the bacterial community composition was conducted using the Bray–Curtis similarity index and MDS analysis. The MDS ordination of 16S tag sequencing data separated the *N. mamillare* samples into three main groups (**Figure 2**). One group corresponded to the CCA sampled from diseased CWPD tissues, a second group included CWBS diseased tissues and the third group had healthy tissues from both control and diseased individuals. There was only one exception with one diseased CWPD sample similar to a healthy community. There was more variability within CWBS disease tissue communities than within CWPD communities (**Figure 2**). In summary, our results show that bacterial communities associated with CWBS were significantly different from the ones found in CWPD diseased tissues (**Table 1**) and they were different from control tissues (ANOSIM, *p* < 0.01), demonstrating that the two syndromes corresponded to different pathobiomes. The pathobiome diversity in patch diseased samples was significantly lower than the diversity of the bacterial community in healthy tissues (**Figure 3**). This finding, together with the observed unchanged diversity of the band syndrome community, is in marked contrast to the patterns that are commonly observed for disease associated microbial communities. Higher diversity has been observed in diseased sponge tissues (Blanquer et al., 2016), and in corals, the yellow band or white plague diseases are associated with an increased number of bacterial taxa (Closek et al., 2014; Roder et al., 2014). These studies have hypothesized that a diverse array of opportunistic bacteria from the surrounding environment colonizes the compromised coral tissues. In the case of CCA, the lower diversity of the CWPD diseased tissues suggests the occurrence of a few specialized pathogens.

**Table 1:** Operational taxonomic units (OTUs) that showed a significant difference in abundance between healthy and diseased tissues (White’s non-parametric *t*-test).

Sample	OTU	Class	Order	Best match	Similarity	Origin or bacterial strain	Reference
Healthy	OTU 62	Alphaproteobacteria	Rhodobacterales	HE574911	98%	*Euprymna scolopes* (squid)	Collins et al., 2012
	OTU 185	Alphaproteobacteria	Rhodobacterales	NR_118329	100%	*Loktanella litorea*	Yoon et al., 2013
	OTU 102	Alphaproteobacteria	Rhodobacterales	AB611495	97%	Sea water	Yoshida-Takashima et al., 2012
	OTU 362	Chloroflexi	Ardenticatenia	KU688646	96%	*Cystoseira compressa* (brown algae)	Mancuso et al., 2016
	OTU 380	Alphaproteobacteria	Parvularculales	EF123358	99%	*Siderastrea siderea* (coral)	Sekar et al., 2008
Band syndrome	OTU 23	Alphaproteobacteria	Rhodobacterales	GU118282	98%	*Diploria strigosa* (coral)	Sunagawa et al., 2010
	OTU 172	Alphaproteobacteria	Rhodobacterales	AY038533	98%	Black band diseased coral	Frias-Lopez et al., 2002
	OTU 239	Alphaproteobacteria	Rhodobacterales	KY577415	98%	Black band diseased *Orbicella faveolata* (coral)	Unpublished
Patch disease	OTU 20	Alphaproteobacteria	Rickettsiales	FJ930418	98%	*Porites lobata* (coral)	Unpublished
	OTU 665	Gammaproteobacteria	Vibrionales	KP329558	100%	*Vibrio tubiashii* Strain T33	Mohamad et al., 2015

*Operational taxonomic units are given together with their best match in GenBank*.

**FIGURE 4:**
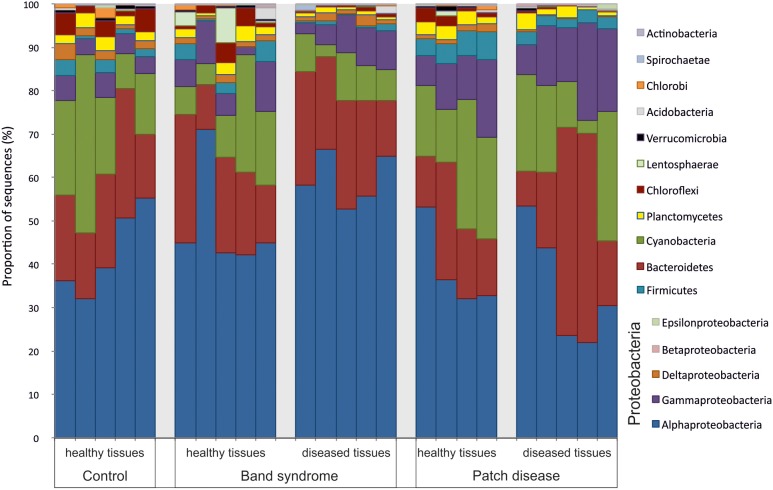
Bacterial community composition at the phylum level in healthy and diseased *N. mamillare* tissues based on 16S rRNA sequences. The most abundant phylum are shown (>0.1% of the sequences).

**FIGURE 5:**
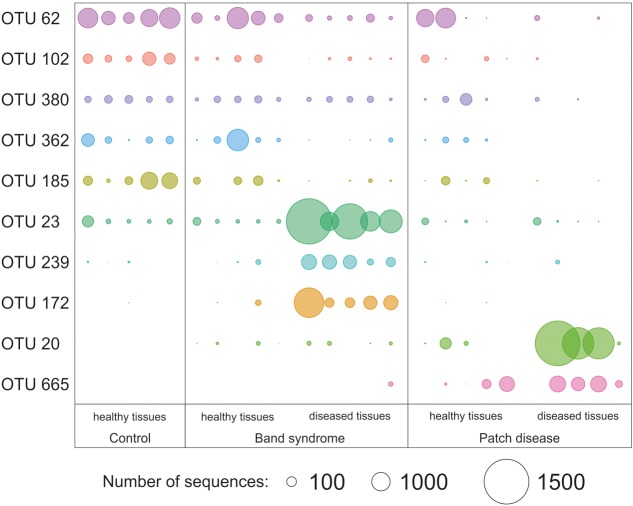
Relative abundance of operational taxonomic units (OTUs) that showed a significant difference in abundance between healthy and diseased tissues (White’s non-parametric *t*-test). Circle sizes are proportional to the number of sequences contained in an OTU.

Diseased CWPD tissues were characterized by a higher proportion of *Bacteroidetes* and *Gammaproteobacteria* (White’s non-parametric *t*-test between control and diseased tissues, *p* < 0.05) (**Figure 4**). At a higher taxonomic resolution, the CWPD pathobiomes showed a significantly higher abundance of a Rickettsiales (OTU 20), similar to an uncultured bacterium isolated from *Porites* coral mucus (White’s non-parametric *t*-test, **Table 1** and **Figure 5**), but without match to any cultured microorganisms. OTU 20 represented up to 13% of the sequences in diseased PD tissues. The CWPD pathobiome also showed a significantly higher abundance of a *Vibrio* sp. OTU (OTU 665, **Figure 5**), which was similar to *Vibrio tubiashii* (**Table 1**), a known pathogen of mollusks often associated with larval vibriosis (Beaz-Hidalgo et al., 2010). This OTU was observed in all the CWPD diseased tissues (**Figure 5**) and represented up to 2% of the sequences in diseased CWPD tissues. High abundances of *Vibrio* were previously found in another CCA species, *P. pachydermum* (21% of sequences), but without correlation to CCA diseases (Sneed et al., 2015). In our study, the vibrio OTU was present in three of the five healthy tissues of the CWPD individuals but was never detected in control individuals (**Figure 5**). This vibrio could thus be the putative agent causing CWPD.

Vibrios are ubiquitous and abundant in the aquatic environment (Thompson et al., 2004). Host endogen factors such as reproduction could play a crucial role in triggering vibriosis (Travers et al., 2009). However, the growth, behavior (Garren et al., 2016) and virulence (Kimes et al., 2012) of pathogenic vibrios in the natural environment are largely dictated by the temperature. The abundance of vibrios has therefore been proposed to represent a microbial barometer of climate change (Baker-Austin et al., 2017). The association between vibriosis and temperature supports our hypothesis of putative pathogenicity of vibrios because CWPD has recently been described as being likely to be temperature-dependent in Curaçao (Quéré et al., 2015b). Together, these results strengthen the hypothesis that CWPD is a temperature dependent disease.

The CWBS pathobiome was dominated by OTUs that belong to the *Alphaproteobacteria* class (**Figure 4**), most of which were *Rhodobacterales* (**Table 1**). The three OTUs, OTU 23, OTU 172 and OTU 239, represented up to 13, 6, and 1.8%, respectively, of the sequences obtained from diseased CWBS tissues (**Figure 5**). We did not identify any potential pathogens in the CWBS community, such as cyanobacteria known to be a causative agent of the black band disease in corals (Casamatta et al., 2012), but *Rhodobacterales* OTUs had close similarity (98%) to sequences found in corals affected by microbial originating white plague or black band disease (Frias-Lopez et al., 2002; Sunagawa et al., 2009). Thus, they could represent opportunist bacteria thriving on dying tissues. They may invade the depleted, diseased CCA cells, which have been shown to contain higher abundances of bioeroders by histological analysis, including boring sponges, helminths, and cyanobacteria (Quéré et al., 2015a). Such as in coral affected by white syndromes disease (Pollock et al., 2017), opportunist bacteria could play a role in pathogenesis and/or serve as a diagnostic criterion for disease differentiation. Further investigations of the pathobiome and/or viral component of the CWBS are required to reveal the causative agent of the disease.

The microbiome associated with control CCA might be specific to the species *N. mamillare.* Only 0.005% of the sequences found in *N. mamillare* were similar to those found in four different CCA species sampled in Belize, including *Titanoderma* textitprototypum and *Hydrolithon boergesenii*, which facilitate coral larval settlement, and *P. solubile* and *P. pachydermum*, which cause lower levels of coral settlement (Ritson-Williams et al., 2014). *N. mamillare* is not known to induce high coral settlements (Quéré et al., 2015b). The presence of a high proportion of cyanobacteria (average of 20% on healthy tissues, **Figure 2**) in *N. mamillare*, *P. solubile*, and *P. pachydermum* feeds the hypothesis that a high abundance of cyanobacteria in certain CCA species could inhibit coral settlements (Sneed et al., 2015). Benthic cyanobacteria, especially those in the order Oscillatoriales, are known to produce potent allelopathic compounds and inhibit coral settlements (Kuffner and Paul, 2004; Kuffner et al., 2006).

In summary, the two different CCA diseases appear to have different causes. The patch disease could be of bacterial origin as shown by the presence of *Vibrio tubiashii*, whereas the origin (i.e., bacterial, viral, or environmental stress) of band syndrome, characterized by opportunistic bacteria, remains unknown.

## Author Contributions

A-LM conceived and designed the experiments, analyzed the data and wrote the paper. PG contributed reagents/material/analysis tools, analyzed the data and wrote the paper. MN contributed materials and wrote the paper. GQ wrote the paper.

## Conflict of Interest Statement

The authors declare that the research was conducted in the absence of any commercial or financial relationships that could be construed as a potential conflict of interest.
